# The skipping behavior of users of music streaming services and its relation to musical structure

**DOI:** 10.1371/journal.pone.0239418

**Published:** 2020-09-30

**Authors:** Nicola Montecchio, Pierre Roy, François Pachet

**Affiliations:** 1 Spotify, Berlin, Germany; 2 Spotify, Paris, France; Beihang University, CHINA

## Abstract

The behavior of users of music streaming services is investigated from the point of view of the temporal dimension of individual songs. Specifically, the main object of the analysis is the point in time within a song at which users stop listening and start streaming another song (“skip”). The main contribution of this study is the ascertainment of a correlation between the distribution in time of skipping events and the musical structure of songs. It is also shown that such distribution is not only specific to the individual songs, but also independent of the cohort of users and date of observation. Finally, user behavioral data is used to train a predictor of the musical structure of a song solely from its acoustic content; it is shown that the use of such data, available in large quantities to music streaming services, yields significant improvements in accuracy over the customary fashion of training this class of algorithms, in which only smaller amounts of hand-labeled data are available.

## Introduction

Since the advent of online music streaming services, people have been able to easily access millions of songs on demand. As a consequence of this abundance, novel listening behaviors have emerged: departing from the passive, concentrated listening practice that is typical of media such as LPs, today people tend to listen to music in a much more frantic way than before.

The central object of interest of this paper is the behavior of users regarding “skipping”: the act of interrupting a song in order to listen to the next song in the music queue (the queue possibly being the rest of the song’s album, a playlist in which the song figures, or a sequence of songs proposed by the recommendation engine of the streaming service).

It is our opinion that skipping is a crucial feature in understanding modern listening behaviors. For the first time in the history of musicology, researchers can systematically collect and analyze massive amounts of data about music listening behavior. Streaming services are only one of many possible contexts in which music is consumed; as such, one must be aware that any observed behaviour is not necessarily generalizable to other situations (e.g., it is safe to assume that skipping behaviour in the context of listening to LPs is radically different from the online music streaming case described in this paper); nonetheless, online streaming services represent nowadays the preferred music consumption mechanism. Statistical analysis of user skipping behavior in time yields indeed fascinating information about how people listen and react to music.

We start by investigating the consistency of skipping behavior with respect to songs, in particular with respect to the case of data collection on different dates and in different regions. We then identify a connection, which to the best of our knowledge has not been observed before, between skip behaviour and musical structure (the segmentation of a musical work into musically relevant sections, such as “intro”, “verse”, “chorus”): listeners are more likely to skip a song right after a change of musical sections. Subsequently, we turn our attention to the task of predicting musical structure from acoustic content (i.e., from the raw audio waveform of a recording), a well known problem in the research community, commonly referred to as *structural segmentation*: we propose a novel approach that makes use of skipping behavior in order to train a prediction algorithm in a semi-supervised way, by exploiting data that can be extracted automatically in large quantities and is directly related to the perception of music by users. We finish by discussing how future lines of inquiries that stem from this study, aimed at a better understanding of user reaction, could potentially lead to the development of compositional tools aimed at improving the reception of music by audiences.

## General statistical aspects of user behavior

**Fig 1:**
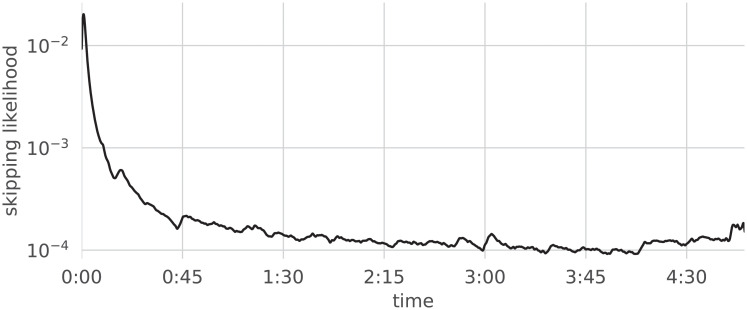
Skip profile of an individual song.

The principal object of study of this paper is the distribution (histogram) of the points in time at which users stop listening to a track, which will be referred to as the track’s “Skip Profile”; a typical example is depicted in Fig 1.

**Fig 2:**
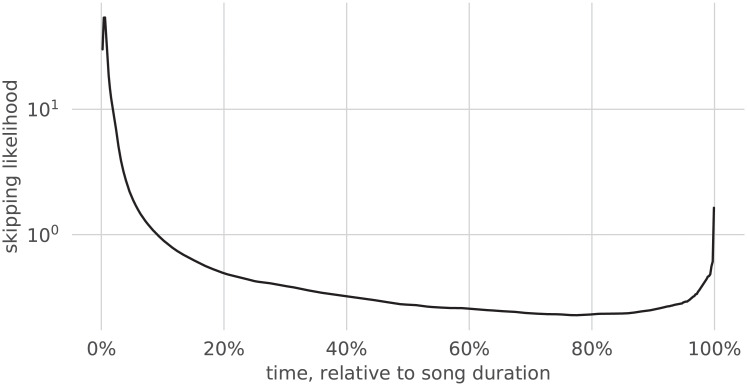
Skip profile of the (aggregated) streams of all songs.

Visual inspection of Skip Profiles suggests a possible interpretation as the superposition of a general trend (pictured in Fig 2, obtained through the aggregation of streaming data over the entire catalog) and a residual signal in which peaks concentrate at specific points in time. In this Section, we investigate the collective behavior of users on the platform, as well as the specificity of skip profiles to songs and their consistency in time and across geographical regions.

### Previous work

Previous research has addressed the issue of modeling music listening; in particular, many works investigated why certain songs become more popular than others. [1] shows that the non-uniformity of subjective preference may be largely explained by so-called *cumulative advantage*, therefore rendering a priori predictions of popularity rather pointless: music hits are inherently unpredictable, due to social pressure. Nonetheless, many works addressing “Hit Song Science” have been published (e.g. [2]), attempting to predict the popularity of a new song based on features automatically extracted from its acoustic content; criticism of such line of works include [3]. All these studies, however, consider songs in their entirety, and do not consider the impact of listeners from a temporal dimension.

A different line of research literature is concerned with the temporal aspect of user responses to musical stimuli. The object of the experiments presented in [4] is to “identify the amount of time necessary to make accurate aesthetic judgments”; in this study the authors argue that such time is around 750ms. Most skipping activity in the context of a commercial music streaming service also occurs in the very first few seconds of listening, thereby suggesting a similar ability of users to very quickly express (negative) aesthetic judgments: preliminary analysis of Skip Profiles [5], averaged on millions of listeners and billions of plays obtained from the commercial streaming service Spotify, identifies a “steep drop off in listeners in the early part of a song, when most listeners are deciding whether or not to skip the song”. It must be pointed out that the context of [5] (and of this paper)—the behavior of generic users in unspecified listening contexts—is radically different from the carefully designed and controlled experimental conditions of [4]; nonetheless, the findings of both are in agreement.

Related work done on large scale music listening behavior data includes the analysis of *scrubbing* behavior [6], that is, the practice of moving the cursor *within* the song in order to search for, and listen to specific parts. The author showed how such data can be used to identify segments of particular interest in songs: instrumental solos, particularly dramatic moments, and, within the genre of electronic dance music, the “drop”.

### Average behavior

**Fig 3:**
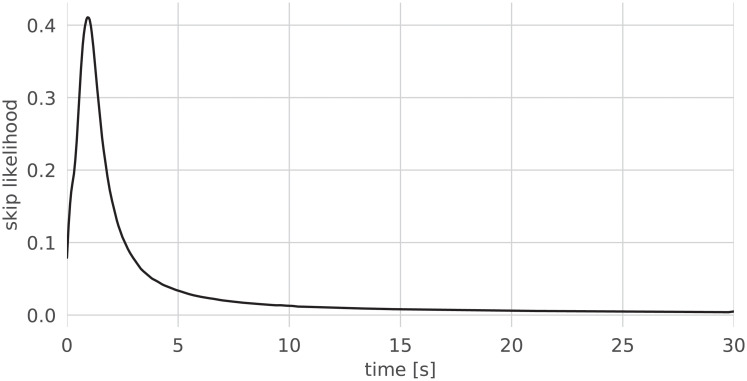
Skipping likelihood at the beginning of a song.

**Fig 4:**
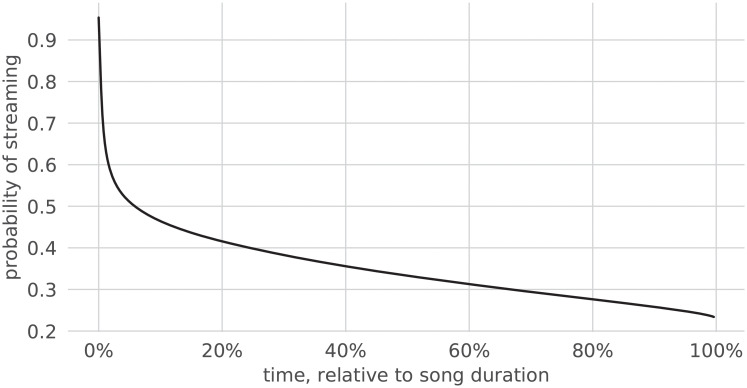
Survival function of skipping with respect to normalized time.

Skipping is an overwhelmingly common behavior of users of streaming services: a quarter of all streamed songs are skipped within the first five seconds, and only roughly half of all songs are listened to in their entirety [5]. That analysis, which dates back to 2014, was reproduced using song streams sampled in August 2018 from Spotify, and its results were confirmed. As can be observed in Fig 2 (in relative time) and Fig 3 (in absolute time), most skips occur indeed at the very beginning of songs; there is also a clear tendency to skip the ending of songs, which often contains several seconds of silence or long fadeouts. Fig 4 shows the survival function of skipping with respect to (normalized) time (i.e., the probability in time that a song is still playing).

### Specificity and consistency

**Fig 5:**
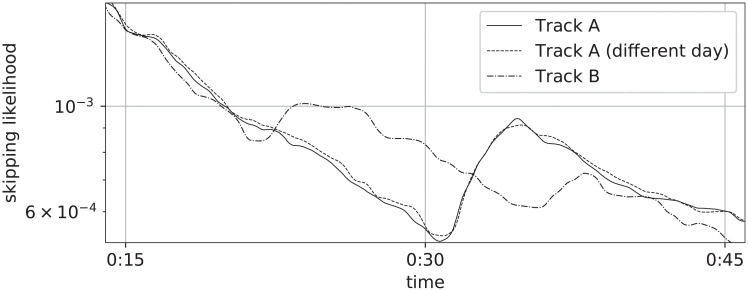
Sections of skip profiles, collected on different days.

Fig 5 shows sections of the Skip Profiles of two songs, for which the data was collected on different days; this particular example shows profiles that are unique to their respective songs and consistent across collection dates. This observation prompts the question of whether this is a general property that holds over a larger collection of songs: can a song be identified from its Skip Profile? Subsequently, the evolution of Skip Profiles is analyzed considering data collected over an extended period of consecutive days and from different geographical regions.

#### Dataset

The dataset used in this Section is composed of 100 popular songs, released in April and May 2018. As of June 1st, 2018, 12 of those songs were among the top 100 most popular songs (in terms of global number of streams), and 40 of them among the top 1000. The songs were selected by an expert musician, with the aim of maximizing variety among genres and avoiding multiple songs from the same artists. Over 3 billion skipping events have been collected from Spotify, spanning a period of three months across all countries in which the streaming service operates; most streaming activity (around 30%) originates from the United States, followed by the United Kingdom, Mexico, Germany. Skipping events have been quantized with a time resolution of 100ms.

#### Specificity of skip profiles

In order to study the specificity of the shape of Skip Profiles with respect to songs, the dataset was processed by considering 30 days worth of observation data, following the release date of each song. The first two minutes of each profile are retained (to account for the variability in length of songs), and independently normalized (to account for the different popularity of the songs); moreover, given that most of the skipping activity occurs in the very first instants, the initial 5 seconds of data are discarded as well. Finally, fragments are slightly smoothed by median-filtering (with a window of 15 samples, corresponding to 1.5s) to de-noise profiles derived from smaller amounts of available streaming data. The resulting vectors have dimensionality 1150.

**Fig 6:**
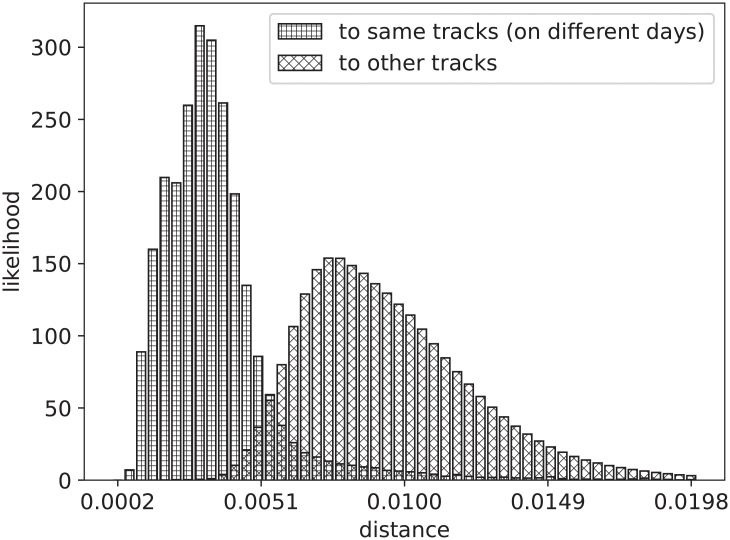
Distributions of same- and different-song distances.

Euclidean distance between the vector representation of Skip Profiles fragments provides a straightforward way to measure specificity: profiles should ideally be closer to other profiles associated to the same song (their streaming data being collected on different days) than to profiles associated to different songs. Fig 6 shows that this is indeed the case, by picturing the distributions of same-song and different-song distances.

This specificity can be further quantified by framing the problem as an Information Retrieval evaluation task [7]. Given a query—a skip profile for a random (track, date) pair—the dataset is sorted by Euclidean distance from the query. Retrieved profiles are considered *relevant* if associated to the query track on different dates, *non-relevant* otherwise. Evaluation can then be carried out using commonly adopted measures, such as Mean Average Precision (MAP) Precision is defined as the fraction of relevant documents among the retrieved ones. Average Precision is the average of the Precision values obtained considering the subsequences of the retrieved documents (sorted by relevance), up to each relevant document in the collection. Mean Average Precision is the mean, over different queries, of the Average Precision value. The range for all these measures is [0, 1].

A random baseline for the experiment on this dataset (obtained by returning a randomly shuffled list of results) yields MAP = 0.014; in contrast, the methodology discussed above yields MAP = 0.886, confirming the hypothesis of specificity of skip profiles.

#### Evolution of skip profiles in time

Let us consider a signal consisting of the differences (in terms of Euclidean distance) between Skip Profiles collected on subsequent days; several possibilities arise: a single S.P. can be selected as reference (that corresponding to the first day being the obvious choice), or the day-to-day difference between the subsequent days can be examined. In this section, the analysis is carried out on one month of data collected following each song’s release date; the release dates on the Streaming service correspond to those of their general availability.

**Fig 7:**
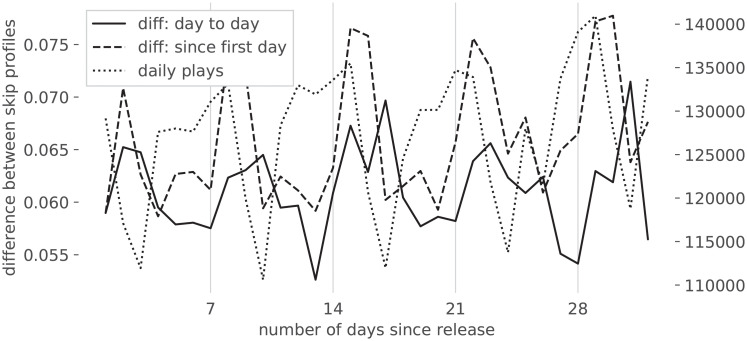
Steady skipping behavior, following a song’s release.

**Fig 8:**
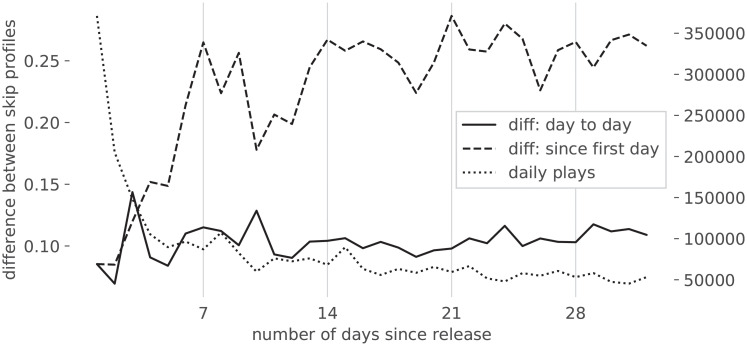
Volatile skipping behavior, following a song’s release.

**Fig 9:**
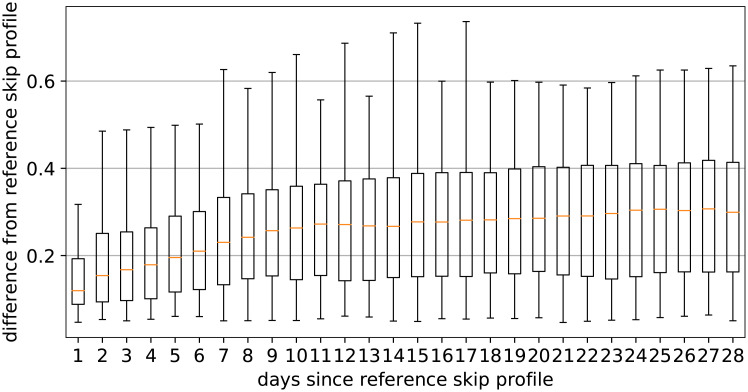
Change, with respect to the release date, over the dataset.

Empirical analysis of the difference signal between subsequent days showed no remarkable anomaly, i.e., there appears to be no point in time in which user behaviour suddenly changes. On the other hand, the evolution of the difference signal with respect to the release date shows more variability, and a determining factor seems to be the trajectory of the number of streams. A steady streaming behavior (Fig 7) is associated to a relatively constant distribution of Skip Profiles over the different days (one can also notice how the weekly listening patterns are reflected in the evolution of the S.P.). On the other hand, the songs exhibiting significant changes in S.P. over different days tend to be those for which the amount of streams changes significantly: Fig 8 shows a declining amount of streams per day, but the behavior for rising amounts of streams is similar. Inspecting the dataset in aggregate, one can better sense the overall evolution: Fig 9 shows, as could reasonably be expected, that in the first two weeks the behavior undergoes the most changes, after which Skip Profiles are fairly stable.

#### Consistency of profiles across regions

The geographical location of users presents an additional way of subdividing user behaviour data. As was the case before, the object of study is the consistency of Skip Profiles, across a different data partitioning scheme.

Streaming data was collected for the same set of songs as in the experiments presented above, and subdivided by country. In order to avoid being influenced by day of release, the data was collected in August of 2018; Skip Profiles composed of less than 100.000 streams were discarded.

As anticipated, empirical observation of Skip Profiles collected across different countries did not suggest any difference among partitions. To validate this hypothesis, we repeated the experiments of the previous Section. The resulting distribution of same- and different-song distances is similar to the one originating from the time-based partitioning; a MAP score of 0.939, in contrast to a baseline of 0.017, was obtained, thus confirming the hypothesis of consistency of profiles across data collection regions.

## User behavior and musical structure

**Fig 10:**
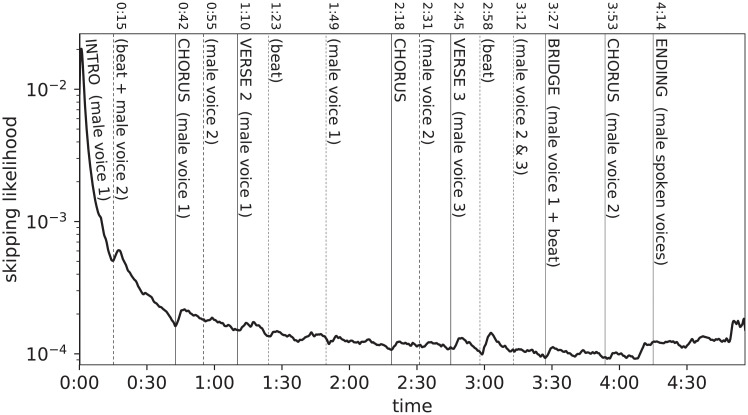
Skip Profile and annotated musical structural boundaries.

Individual Skip Profiles exhibit deviations from their aggregated trend (Fig 2) in a way that is closely related to musical structure. Fig 10 shows a song’s Skip Profile, overlayed with musical structural boundaries. It is visually evident that section boundaries are commonly followed by surges in the likelihood of skipping.

This Section investigates the correlation between user skipping behavior and musical structure. To the best of the authors’ knowledge, such correlation has not been observed before. It is first shown that the location of musically relevant boundaries can be predicted directly from skip behavioral data. The accuracy of the prediction is then evaluated against a collection of existing algorithms, developed in the field of Music Information Retrieval, that predict musical section boundaries from the content of recordings (i.e., the acoustic waveforms). Skipping behavior data is finally exploited to automatically generate large quantities of training data (otherwise very cumbersome and costly to create manually) for content-based Machine Learning algorithms, and the accuracy of algorithms trained using such data is evaluated against the customary way of training, as well as multiple other baselines.

### Related work

Content-based musical structure segmentation has been a central research subject in the field of Music Information Retrieval for many years. A comprehensive overview of the topic [8] defines the main goal of a structural segmentation algorithm: “to divide an audio recording into temporal segments corresponding to musical parts and to group these segments into musically meaningful categories”. The input of such algorithms is the audio content of a music recording (i.e., a digital encoding of the acoustic waveform), the output is a subdivision of the song into (usually) non-overlapping windows; some algorithms associate a label to each section, which can be descriptive (such as “bridge” and “chorus”) or just identifying of repetitive sections (e.g., “A”, “B” and so on). As discussed in the above-mentioned paper, most algorithms can be categorized into three conceptual approaches, based on *repetition*, *novelty* and *homogeneity*, that is the identification of, respectively, recurring patterns, transitions between contrasting parts, and contiguous sections that are consistent with respect to some musical property; algorithms typically make use of features that attempt to take into account musical characteristics such as melody, harmony, rhythm, and timbre. A more recent overview of the State of the Art in the field can be found in [9].

Research on the topic has long been hindered by the lack of sizeable amounts of expert annotations—the manual segmentations and labelings of recordings done by musically-competent individuals—because of its time-consuming nature, coupled with the legal retrictions involved in to sharing copyrighted recordings. Initiatives such as the SALAMI dataset [10] attempt to fill this need by providing a relatively large (several hundreds) source of annotations for recordings, many of which are in the public domain.

An alternative approach to deal with the aforementioned issues is the release of the algorithms themselves as open source software: MSAF [11] is the leading effort in that regard within the Music Information Retrieval community, and couples that goal with an evaluation framework; the latter is in turn based on MIReval [12], a reference implementation of a large set of common music-specific IR evaluation metrics. The remainder of this paper makes use of the following algorithm implementations borrowed from MSAF: cnmf [13], foote [14], olda [15], scluster [16], sf [17]. Furthermore, another reference algorithm is given by the The Echo Nest Analyzer, based on [18], whose results can be accessed through Spotify’s public API.

Newer approaches, based on more recent Machine Learning techniques, include [19] and [20], that achieve State of the Art accuracy in prediction by making use of a Convolutional Neural Network architecture.

### Correlation of skip profiles and musical structure

**Fig 11:**
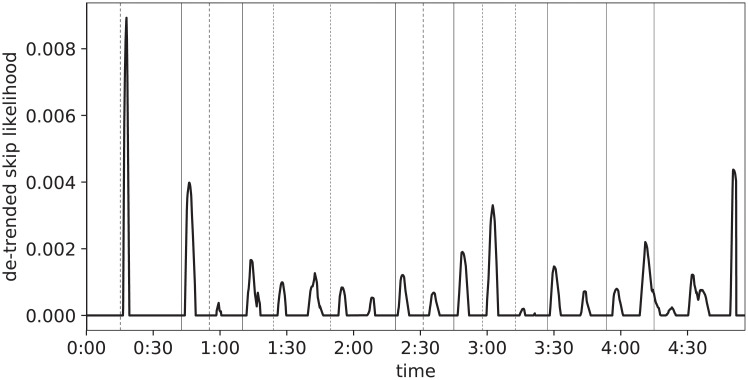
De-trended Skip Profile and musical structural boundaries.

In order to emphasize the regions of a Skip Profile that depart significantly from its overall course, a de-trending algorithm is applied, the results of which are depicted in Fig 11. The core of the procedure is a deliberately simple heuristic, aimed at isolating the general trend of the profile: median filtering if first applied (with filter size 19, corresponding to 1.9s), then a fifth-order Butterworth lowpass filter with 0.03 cut frequency; this trend is subsequently subtracted from the original signal and the residual signal is rectified.

This graphical representation makes it even clearer that surges in skips regularly follow section boundaries, after a short delay. Such delay was estimated, via a grid-search procedure, to be around 3.5s (corresponding to the delay yielding the highest accuracy according to the evaluation procedures detailed below, in Section “Evaluation”). The delay can be interpreted as the sum of two components: the time it takes a user to realize they do not want to be listening anymore to the current song—triggered by the crossing of a section boundary—and the time spent interacting with the reproduction device (tapping or clicking on the “next” button).

**Fig 12:**
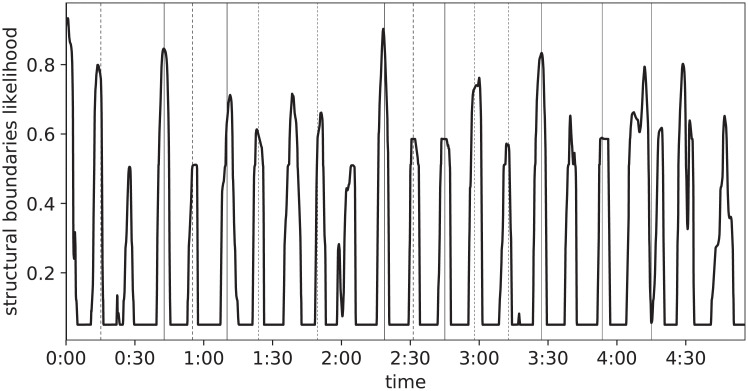
Likelihood of structural boundaries, predicted from the skip profile.

To prove the existence of a relationship between user behavior and musical structure, we show that the latter (specifically, the location of section boundaries) can be predicted from the former (a Skip Profile). To this end, a compact Neural Network is trained on short segments (30s) of Skip Profiles; its objective is to predict whether the central location of each particular segment in the original signal falls close enough (within 1s) to a section boundary. It is sufficient to annotate only a few dozen songs to obtain satisfactory performance, and the results of such procedure can be observed in Fig 12, clearly showing the strong relation between music structure and user behavior (a quantitative evaluation is carried out below).

### Training acoustic content-based structure prediction models using behavioral data

An existing algorithm from the Music Information Retrieval literature is exploited to demonstrate the effectiveness of using Skip Profiles for training content-based section prediction algorithms.

**Fig 13:**
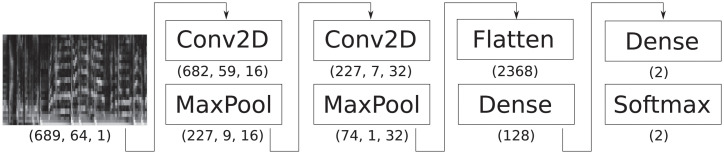
Architecture of the network for predicting structural boundaries from audio.

The algorithm, detailed in [19], is based on a straightforward, well understood Convolutional Neural Network architecture. Originally designed in [21] for the task of *onset detection*—the task of detecting the instants at which musical events, such as individual notes or chords, occur—the architecture of the model for structural prediction is unchanged, except for the longer input ranges considered. The model is at its core a binary classifier that predicts the likelihood of the presence of a section boundary in the center of an audio excerpt. A diagram of the network architecture is shown in Fig 13.

The input of the network is a segment of an audio recording, for which a *Mel-Spectrogram* is extracted; the latter a common transformation in the music signal processing literature [22] consisting of a spectrogram (a time-frequency representation obtained by concatenating the individual magnitude of the Fourier transforms of short, overlapping excerpts of the signal) whose frequency bands are then warped according to a perceptual (“Mel”) scale.

The output is a scalar, whose value depends on the distance of the closest section boundary from the center of the audio input. In [19] a strategy known as *target smearing* is employed, which accounts for the inaccuracy of ground truth boundary annotations: during the training phase, only the excerpts centered on a frame that is sufficiently close to a section boundary are presented to the network as positive examples, and their target value is the weight of a Gaussian kernel, evaluated at the distance in time between the center of the excerpt and the closest section boundary.

Fine-grained annotation of individual songs, in terms of the locations of structural boundaries, is used for training this class of algorithms: such annotation is carried out manually in a very laborious and time consuming way. In order to exploit of the correlation between Skip Profile and structure, the model described in the Section above is used to generate large quantities of training data. From the model predictions, only regions of very high and very low boundary likelihood are retained; empirical observation suggests that false positive samples are generated more frequently than false negative ones, as can be seen in Fig 12.

The trained network can be used to create a prediction by repeatedly applying it to adjacent, overlapping segments of a recording. The output is a vector whose length is proportional to the length of the input recording. In order to extract a discrete set of time instants (the estimated structural boundaries) from it, a peak-picking procedure is used: a point in the likelihood vector is considered a peak if it is a local maximum and is far enough (a few seconds) from other peaks; a threshold is set to half the value of the likelihood of the third-highest peak.

**Fig 14:**
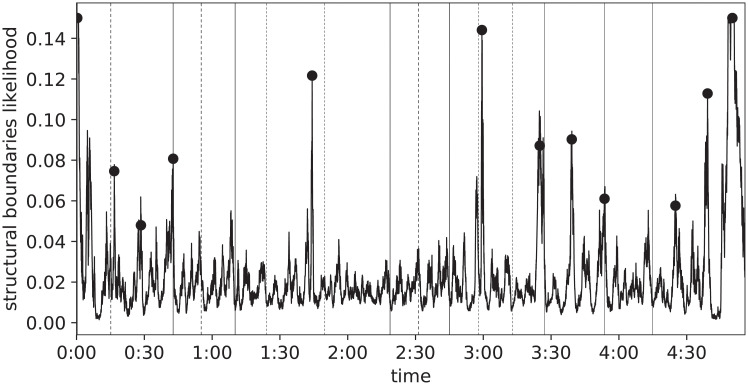
Likelihood of structural boundaries, predicted from the acoustic content.

An example prediction is pictured in Fig 14, along with the estimated boundaries marked with circles.

### Evaluation

The evaluation of segmentation algorithms is customarily formulated in terms of (approximate) overlap between two sets of instants in time: the actual timings of section boundaries (as annotated by an expert curator) and those predicted by an algorithm. Each prediction is considered a *hit* if it falls within a certain range from any reference boundary timing, a *miss* otherwise.

It is common in the literature to consider two such interval sizes, of 0.5 and 3s, and to use F-score F-score is defined as the harmonic mean of Precision and Recall; the latter is defined as the fraction of relevant instances that have been retrieved over the total amount of relevant instances. as the preferred evaluation measure. The evaluation of segmentation algorithms forms the subject of [23], in which it is argued that an appropriate weighting of the F-Score factors (as opposed to the default unit weighting) is a measure that better corresponds to human perception; the results presented below conform to such weighting scheme.

#### Datasets

The experiments were carried out making use of several datasets. No song belongs to multiple sets.

- SALAMI [10]: already mentioned above, is comprised of the annotations for 1164 songs; however the recordings for only a subset of these songs are publicly available, yielding 376 useable recording/annotation pairs. In case of multiple annotations per song, only the first one was considered. These songs are not commercial recordings, hence no associated user behavior data is available.
- TOP100: the dataset consists of the one hundred most popular songs (by number of streams, globally) on Spotify as of April 1, 2018. The structure of each song was manually annotated by a single annotator (a professional musician). The skip profiles for these songs, derived from roughly 1 billion streams, were collected over the month of May 2018.
- SP20k: a dataset of the Skip Profiles for roughly 20k songs; only songs with at least 100k streams over a period of 3 months are retained, totalling about 81 billion streams.

In the TOP100 dataset, two types of boundaries were annotated: “structural”, that only includes proper structural boundaries (such as “Intro”, “Chorus”, “Verse”), and “extended”, that includes additional significant events happening in the song (e.g., the entrance of a different singer within a musical section). In Fig 10 the two types of boundaries can be observed (solid line for structural boundaries, dashed line for non-structural, extended boundaries). Non-structural extended boundaries often occur half-way through a musical section: a typical case is a verse constituted by the repetition of a musical phrase, in which the second occurrence is characterized by the presence of additional (usually percussive) backing instruments.

#### Results

Table 1 reports the results of the evaluation, in terms of Weighted F-Score for the Hit-Rate metric, of several algorithms on the TOP100 dataset.

**Table 1:** Hit rate (weighted F-Score) for several algorithms, on the TOP100 dataset.

Algorithm	boundaries:	Structural		Extended	
	“hit” window size:	0.5s	3s	0.5s	3s
baseline		0.053	0.273	0.079	0.372
foote [14]		0.085	0.415	0.127	0.511
olda [15]		0.158	0.504	0.217	0.609
scluster [16]		0.126	0.354	0.170	0.474
sf [17]		0.106	0.425	0.165	0.502
ten [18]		0.152	0.536	0.190	0.607
skipprofile-nn		0.245	0.630	0.278	0.636
audio-nn-salami [19]		0.226	0.464	0.287	0.522
audio-nn-skip-profiles		0.259	0.560	0.307	0.638
audio-nn-finetune		0.311	0.575	0.373	0.658

The baseline entry refers to a trivial estimator, which always predicts boundaries at fixed regular intervals; the particular spacing (12s) has been determined through a grid-search procedure, in order to obtain the highest possible F-Score. This particular baseline methodology mirrors that of [19], in which the reported values are however significantly higher (0.13 and 0.33, for window sizes of 0.5s and 3s, respectively); the large difference is attributable to the different evaluation dataset used, and to the weighting scheme applied to the F-Score metric.

Next, the foote, olda, scluster, and sf entries refer to algorithms that have an open-source implementation provided by MSAF https://github.com/urinieto/msaf, and ten refers to a commercial algorithm (The Echo Nest, now part of Spotify). In all of these instances, the default parameters (if any) were utilized.

The entry skipprofile-nn refers to the section boundary predictor described above, that takes Skip Profiles as input; it validates the fundamental thesis proposed in this paper, namely, that musical structure and user behavior are correlated. The model is a straightforward Feed-Forward Neural Network operating on 30s input windows of Skip Profile signals (300 frames), and consists of a cascade of 5 dense layers, each with 0.5 dropout probability, 64 hidden units, and Exponential Linear Unit activations; the network is trained to minimize a cross-entropy loss, using the ADAM optimizer (in its default parametrization). For each song in the training dataset, 21 positive example per boundary are selected, whose center is at regular intervals between -1s and 1s away from the section boundary, and 1000 negative examples are chosen at other random locations. The reported F-Score values are computed through 5-fold cross-validation.

The entry audio-nn-salami represents the model and training procedure described in [19]. The original paper presents several variants of the model, the largest of which was adopted. Attempts were made to re-implement the described model as closely as possible; because of the smaller training set available to us (this algorithm is trained using the publicly available, 376-songs subset of SALAMI as detailed above, as opposed to a dataset of 1220 annotated recordings available to the authors of the original paper), and in order not to be hindered by possible misinterpretations of the description, several experiments were performed to find the best settings for the model, so as to provide the highest baseline. The resulting network operates on spectrogram windows of size 689 (equivalent to 32 second windows of audio) by 64. After log-scaling, the signal goes through two convolutional layers (respectively of 16 and 32 channels, and filter sizes 8 by 6 and 6 by 3) with Leaky Rectified Linear Unit (ReLU) activations, each followed by a max-pooling layer of size 3 by 6; finally the signal goes through a dense layer (of 128 units, also with Leaky ReLU activations) and undergoes softmax scaling. As before, the network is trained to minimize a cross-entropy loss, using the ADAM optimizer (in its default parametrization), for 10 thousand iterations with a batch size of 8.

The entry audio-nn-skip-profiles is again the audio-nn-salami model, however trained using solely data derived from user behavior (the SP20k dataset), as described above. The exact same parametrization and learning strategy as before were used when performing this experiment, in order to restrict the difference in outcome to the data source. The reported results show that it is indeed possible to achieve state of the art performance using large quantities of unannotated training data.

The final entry, audio-nn-finetune, obtains the best performances by combining both sources of training data. The audio-nn-skip-profiles model, discussed above, is taken as the starting point; subsequently, it is fine-tuned by training the last layer using the SALAMI dataset. This allows the model to exploit both Skip Profiles and manually assembled sources: the former, a source of unreliable data available in large quantities, is used to learn a robust feature representation, while the latter is used to efficiently exploit the high-precision nature of a small, hand-curated dataset.

It should be taken into consideration that the nature of the Salami dataset is radically different from that of SP20k, in the first place regarding recording quality, which is much lower for Salami on average. Unfortunately, the two aspects are in opposition: for songs to have enough skipping data points, they must be popular, and the production quality of such popular songs is for the most part extremely high; the automated creation of a large-scale dataset with similar characteristics as Salami would be a very complicated matter. The large improvement in accuracy nonetheless obtained by the audio-nn-finetune model via fine-tuning, despite the different nature of the datasets, can be attributed to the fact that the nature of the music in the two datasets with respect to musical structure is rather similar, most songs being characterizable as popular, commercial music.

## Discussion

The original goal of the study was to leverage massive amounts of fine-grained information collected by streaming services, in order to better understand how songs are received by their audience. A better understanding could in principle be used to inform the design of novel compositional tools that take into account a model of the listener grounded in actual music consumption data.

Through the investigation of large-scale user behavior, a previously unknown correlation between skipping and musical structure has emerged. A joint analysis of the distribution of musical sections and the overall trend of skip profiles, analyzed across a large catalog, is the object of future research inquiries. It is the authors’ opinion that such analysis has the potential to pave the way for the design of tools that can leverage and anticipate user response in order to provide useful guidance to creators.

An additional future research direction is the modeling of the response of individual users to the songs to which they listen. The effect that musical features—such as genre, mood, or instrumentation, just to name a few—have on the reception by users is a well studied problem in the literature and in industry, and closely tied to the field of Recommender Systems. The authors are however not aware of existing work that attempts to jointly exploit content-based Music Information Retrieval methods and user modeling with fine-grained temporal user information to influence recommendation, and believe that this study can provide a starting point for such future research directions.
